# Sex-Specific Variation in Metabolic Responses to Diet

**DOI:** 10.3390/nu16172921

**Published:** 2024-09-01

**Authors:** Reya R. Andrews, Kayla R. Anderson, Jean L. Fry

**Affiliations:** 1 Department of Pharmacology and Nutritional Sciences, College of Medicine, University of Kentucky, Lexington, KY 40506, USA; reya.andrews@uky.edu; 2 Center for Muscle Biology, College of Health Sciences, University of Kentucky, Lexington, KY 40536, USA; kan266@uky.edu

**Keywords:** sexual dimorphism, dietary patterns, metabolic health, type 2 diabetes, insulin, glucose tolerance, body composition, cardiometabolic, sex difference, diet

## Abstract

Suboptimal nutrition is a leading cause of cardiometabolic disease and mortality. Biological sex is a variable that influences individual responses to dietary components and may modulate the impact of diet on metabolic health and disease risk. This review describes findings of studies reporting how biological sex may associate with or affect metabolic outcomes or disease risk in response to varying dietary macronutrient content, Mediterranean diet, Western diet, and medical very low-calorie diet. Although few dietary interventions have been specifically designed to identify sex–diet interactions, future studies improving understanding how sex influences dietary responses could inform precision nutrition interventions for disease prevention and management.

## 1. Introduction

It is well recognized that diet plays a major contributing role in today’s major chronic disease epidemics, accounting for an estimated 45% of cardiometabolic fatalities [1]. Dietary patterns profoundly affect insulin sensitivity, metabolic syndrome, obesity, cardiovascular disease, and type 2 diabetes risk (T2D). For example, the Western diet, characterized by high refined carbohydrates, saturated fats, and processed foods, is associated with increased insulin resistance and poor glucose tolerance, contributing significantly to the rising prevalence of T2D [2,3,4,5,6]. In contrast, the Mediterranean diet—rich in fruits, vegetables, whole grains, and healthy fats like olive oil—enhances insulin sensitivity and improves glucose metabolism [7,8], thereby reducing T2D risk. At the same time, caloric restriction, even without specific macronutrient adjustments or food group specifications, improves insulin sensitivity and lowers fasting glucose concentrations [9]. Intermittent fasting and time-restricted feeding patterns are linked to reduced insulin resistance and better glucose regulation [10,11]. 

Evidence indicates that biological sex affects response to diet-based interventions. Sexual dimorphism, the phenotypic differences between males and females of the same species, extends beyond anatomical and physiological characteristics to include responses to dietary intake and metabolic processes [12]. It is established that some nutrient recommendations vary between males and females. For instance, iron requirements are higher in females due to menstrual losses [13]. Biological factors underpinning sexual dimorphism in nutritional needs and response to nutrient intake may include sex chromosome dosage [14], hormonal differences [15,16,17], body composition [18], nutrient requirements [19,20], disease susceptibility [21,22], and reproductive stage [23,24], among others. 

Defining sexual dimorphism in diet response is crucial for developing precision dietary recommendations to optimize metabolic health and prevent or manage chronic disease. Here, we seek to describe how biological males and females respond differently to variable macronutrient, Mediterranean, Western, and medical very low-calorie diets (VLCD), with an emphasis on cardiometabolic outcomes. A comprehensive review of underlying mechanisms and studies including preclinical models is outside the scope of this review, but MacArthur and Mitchell recently published an excellent review of sex differences in health span and lifespan in response to several dietary interventions in model organisms [25]. Here, we emphasize sexual dimorphism in response to diet reported in original research including human participants.

## 2. Methods

To write this narrative review, we conducted a literature search using PubMed to identify studies examining sex-specific responses to dietary intake. The search terms included combinations of keywords such as “sex-specific response”, “sex differences”, “gender differences”, “X chromosome”, “Y chromosome”, “lipid metabolism”, “body composition”, “metabolic disease risk”, “type 2 diabetes”, “insulin resistance”, “Mediterranean”, “plant-based”, “vegan”, “vegetarian”, “ketogenic”, “Western”, “diet”, “dietary intake”, “nutritional response”, “metabolism”, “men”, “women”, “hormones”, “OGTT”, and “nutrition”. Studies were also gleaned from citations of the articles identified through the PubMed searches.

Studies included in the review met the following criteria: prospective cohort studies, clinical trials, preclinical studies, or meta-analyses of these study types; examined responses to dietary intake; presented data separately for males and females; reported quantitative data on metabolic, physiological, or health-related outcomes; published in peer-reviewed journals. Studies were excluded if they relied on cross-sectional or retrospective designs, focused on non-dietary interventions or predictors, or were unavailable in the full text. The titles and abstracts of identified articles were screened using inclusion/exclusion criteria. Full-text versions of potentially relevant articles were retrieved and assessed for eligibility. All articles we identified that reported clinical trials designed to test sex-based differences in dietary response were included. All authors reviewed the full texts of manuscripts highlighted in the tables, and discrepancies in study interpretation were resolved through discussion.

Data extracted included study characteristics, participant demographics, dietary intervention or pattern details, and sex-specific outcomes. While the review included human studies, findings in preclinical models have been incorporated for comparative purposes. This narrative review outlines the findings of studies that examine how biological sex might influence or be linked to metabolic outcomes or disease risk in response to different dietary macronutrient content, the Mediterranean diet, the Western diet, and VLCD.

## 3. Macronutrient Ratios and Quality

### 3.1. Carbohydrates

Data from cohort studies and completed clinical trials were utilized to explore whether dietary patterns emphasizing varying macronutrient ratios or macronutrient sources affected men and women differently (Table 1). Utilizing substitution models and data from the European Prospective Investigation into Cancer and Nutrition (EPIC)–Potsdam cohort study, investigators sought to determine whether certain macronutrients, or their subgroups, predicted development of T2D over 8–10 years in middle-aged men (*n* = 9702) and women (*n* = 15,365) [26]. At baseline, men in the highest quintile of energy intake from carbohydrates had lower BMI and waist circumferences; however, this relationship was not seen in the women. While carbohydrate intake did not affect development of T2D in women, men with the highest carbohydrate intake were less likely to develop T2D over the follow-up period; however, this finding was lost with adjustment for BMI and waist circumference. When including both male and female participants, substitution modeling showed that replacing 5% energy contribution from protein or polyunsaturated fatty acids (PUFA) with carbohydrates reduced risk of developing T2D, and the association was stronger in participants without obesity. In fully adjusted, sex-stratified analyses, the only significant finding was that replacing 5% energy contribution from protein with carbohydrates reduced T2D risk in men. These results suggest, for example, that when men consume a diet with 2500 kcal and replace 5% of energy from protein (approximately 31 g) with carbohydrates, their T2D risk may be attenuated. 

Some evidence indicates that women are especially susceptible to chronic disease associated with a high-glycemic-index (GI) dietary pattern [27,28]. One group sought to determine whether glucose and insulin responses to meal challenges were different between men and women when assigned to either a low- or high-GI Mediterranean diet for 12 weeks [29]. Using data from the MEDGI-Carb trial, a clinical trial comparing high- and low-GI Mediterranean diets, 156 middle-aged and older adults with a BMI ≥ 25 and a high waist circumference were included in a secondary analysis. The two diets called for equal amounts of carbohydrates and fiber, differing only in starch sources. In both diet groups, half of the carbohydrates were from fruits, vegetables, and dairy. The low-GI group (<55) were additionally provided with pasta, brown rice, flatbread, all bran, and wheat bread plus rye and seeds, while the high-GI diet group (>70) were given jasmine rice, potatoes, couscous, wholegrain bread, and biscuits. Energy intake was eucaloric to maintain stable bodyweight. Post-intervention, participants underwent two meal challenges with concurrent observation blood sampling for 8 h, and investigators determined average plasma glucose and insulin concentrations. While there were no notable differences in insulin secretion post-intervention compared with baseline, women on the high-GI diet showed considerably higher 8 h mean glucose concentrations in response to the meal challenges compared with baseline values, and these differences were not observed in the men assigned to the high-GI diet. These findings suggest that women may not tolerate chronic consumption of rapidly absorbed carbohydrates as well as men. On the other hand, a recent meta-analysis evaluating the effects of GI and glycemic load on cardiometabolic health concluded that both sexes show greater risk of T2D development with a high-GI diet independent of overall dietary pattern [30]. That said, women showed greater T2D risk in association with a high-GI diet compared with men, after controlling for the dietary instruments’ correlation coefficients for carbohydrates, ethnicity, and duration of follow-up [30].

Similarly, using dietary data from the Japan Public Health Center-Based Prospective Study, investigators sought to determine whether intake of several common dietary grain products—including rice, noodles, and bread—affected the risk of T2D development in men (*n* = 25,666) and women (*n* = 33,622) 45–75 years of age [31]. Women who consumed three bowls (420 g) or more of white rice each day showed an increased risk for T2D; however, the men did not show increasing T2D risk with greater white rice intake. The observed risk increase in women was greater among non-obese women, lending credibility to the idea that the dietary factor, rather than overall weight, affected T2D risk. Since the analysis was specific to white rice, that study further supports the idea that refined grain products are more deleterious to women’s metabolic health compared with men. The risk in women was attenuated among those with high levels of physical activity, showing the importance of considering the impact of physical activity on diet–disease relationships. Another analysis of the same cohort study showed high intakes of total sugar, fructose, and starch were associated with risk of developing T2D in women, but men did not show increased T2D risk with high consumption of these refined dietary carbohydrates [32]. A recent meta-analysis of cohort data from eight Asian countries (*n* = 256,818) showed a higher relative risk of T2D development in women (1.58) with high white rice intake compared with men (1.30); however, the interaction effect was not significant [33]. That said, that meta-analysis did not consider the potential impact of physical activity on the relationship.

A study of acrylamide intake and incidence of T2D in a cohort of adult participants without T2D (*n* = 6022) from the Tehran Lipid and Glucose Study suggested that high intake of processed foods high in carbohydrate may be especially deleterious for women [34]. Acrylamide is a chemical compound that is formed from carbohydrate and the amino acid asparagine through the Maillard reaction during the high-temperature cooking of carbohydrate-rich foods, such as potato chips, French fries, and bread. Acrylamide intake was not associated with the incidence of T2D in the overall study group after adjusting for potential confounders; however, in a subgroup analysis, women in the highest quartile of acrylamide intake showed a significantly increased risk of developing T2D compared with the lowest quartile, indicating that high intake of processed carbohydrates may be pose a greater risk for women.

The combined findings suggest that women are more susceptible to glucose intolerance in response to chronic consumption of large amounts of rapidly absorbed and refined carbohydrates.

### 3.2. Lipids

Women may better tolerate less healthful dietary sources of lipids. To evaluate whether biological sex affects postprandial lipemia, investigators instructed young, healthy participants (*n* = 10) to consume a self-selected low-fat, low-cholesterol diet and after another two weeks, a self-selected high-fat, high-cholesterol diet [35]. On the high-fat diet, when compared with the low-fat diet, both men and women reported comparable and substantial increases in energy, cholesterol, and total fat intake (approximately 40% increase in total kcal, 1000 mg increase in daily cholesterol, and shift in fat from 30% to 40% of total energy). While fasting circulating cholesterol concentrations increased similarly for both sexes following the high-fat diet, only men showed a significant postprandial increase in triglycerides following a weight-adjusted challenge meal of tea, rolls, ham, and liquid cream. 

Though estrogen affects lipid metabolism and circulating lipid concentrations [36], there is evidence that boys and girls may respond differently to modulations in dietary fat even before overt sex hormone concentrations vary between the sexes. The Special Turku Coronary Risk Factor Intervention Project implemented a low-saturated fat, low-cholesterol randomized diet intervention in children from birth to three years of age and compared these children with a control group (*n* = 1062) [37]. The intervention group’s families received continuous dietary counseling, which resulted in significantly lower total cholesterol concentrations without adverse effects on growth or development. The investigators noted a significant interaction between sex and treatment group in terms of baseline-adjusted total cholesterol, then followed up with an analysis of data disaggregated by sex. The intervention had a more pronounced effect on boys and showed baseline-adjusted total cholesterol concentrations that were approximately 6% lower than the control group (*p* < 0.0001). Baseline-adjusted total cholesterol concentrations were not different between girls in the intervention and girls in the control group (3% lower in intervention girls; *p* = 0.089). Since boys and girls show different responses to low-fat diets at an early age, sex-based differences in response to dietary lipids may not be explained entirely by metabolic differences induced by sex hormones.

Studies including rodents testing high-fat diets typically show that male rodents exhibit hyperphagia, increased bodyweight, and poor metabolic outcomes compared with females [38,39,40]. Estrany et al. conducted a trial that investigated the impact of a high-fat diet on glucose tolerance, insulin signaling, and lipid catabolism in Wistar rats [38]. Male and female rats were assigned to either a standard diet (2.9% w/w fat) or a high-fat diet (30% w/w fat) for 14 weeks. Male rats consuming a high-fat diet showed greater adiposity and impaired glucose tolerance with concomitant decrease in *Pparg* compared with male rats consuming a standard diet. *Pparg* is a nuclear receptor with regulation activity promoting insulin sensitivity and reducing inflammation associated with T2D and atherosclerosis. When female rats consumed a high-fat diet, they remained glucose tolerant and showed higher expression of *Pparg* transcripts and carnitine palmitoyltransferase 1 (CPT1) protein. CPT1 is responsible for transporting fatty acids into the mitochondria for beta-oxidation, so these findings suggest that females adapt to high-fat diets in part by upregulating fatty acid oxidation.

Elzinga et al. investigated the effects of a high-fat (60% fat) or a standard diet (10% fat) on insulin resistance and peripheral neuropathy in male and female mice and found similar sex-based variation [40]. While both sexes experienced similar nerve-conduction deficits and nerve fiber loss, male mice gained weight more rapidly than females throughout the course of the study up until 36 weeks, and females also displayed delayed insulin resistance [40]. Huang et al. fed young mice either a standard diet (10% fat) or a high-fat diet (45% fat) for 5 weeks and measured food intake, energy expenditure, weight, and fat mass over this period [39]. Males on the high-fat diet had significantly higher weight and adipose tissue after 5 weeks compared with males assigned to the standard diet, while females consuming the high-fat diet had significantly greater weight, but not fat mass, compared with females on a low-fat diet. Males on the high-fat diet had lower energy expenditure during the light [sleeping/inactive] phase compared with high-fat diet-consuming females. While both male and female mice consuming the high-fat diet showed a lower RER than their same-sex low-fat diet counterparts, the area under the curve difference over 24 h appeared more substantial in the females compared with the males, but statistics were not completed to test that difference. These RER data appear to reinforce the molecular observations showing that female rodents consuming a high-fat diet show greater expression of enzymes associated with lipid catabolism compared with females consuming a lower-fat diet. Females may tolerate a chronic high-fat diet better than males due to their greater ability to upregulate the use of fat as a fuel source.

### 3.3. Protein

The role of dietary protein in metabolic health is under great scrutiny [41]. While some dietary intervention studies show the benefits of protein or amino acids for improved metabolic outcomes [42], energy expenditure [43], retention of lean mass during weight loss, and enhanced satiety [44,45,46], others have associated protein intake with higher risk for morbidity and mortality from common cardiometabolic diseases [41,47]. Recently, convincing preclinical data have shown that high protein and amino acid intake causes metabolic impairment in rodents, while dietary restriction of protein and certain amino acids (e.g., isoleucine and valine) promotes improved metabolic outcomes mimicking calorie restriction [47,48]. Reliable human data on the metabolic effects of protein intake are difficult to identify for several reasons. Many human studies have tended to focus on modifying carbohydrates or fat while keeping protein consistent at around 10–16% of overall energy intake. The 10% value is the bottom of the dietary reference intakes acceptable macronutrient distribution range for protein, while the 2017–2020 pre-pandemic What We Eat in America data show that the US population consumes approximately 14–16% of their energy from protein, a level which has been consistent in recent decades. 

The effect of protein intake on health is further complicated by the difficulty of standardizing intake, particularly in cohort and cross-sectional studies. Though protein needs and recommendations are often given standardized to weight (e.g., the RDA of 0.8 g/kg), standardizing protein intake to weight in cross-sectional or cohort studies leads to the reporting of specious relationships between high protein intake and metabolic health [49]. Analyses using protein intake standardized to weight are often published, but methods standardizing to total energy intake or ideal or fat-free bodyweight are less likely to yield spurious results [49]. Protein research is further complicated by a lack of consistency in the definition of high, moderate, or low protein and the level of compliance with the assigned diet [50]. Finally, studies of protein modulation within a lower energy diet are complicated by the fact that absolute protein intake on a weight maintenance diet and a higher protein diet, defined as a percent of macronutrients, may be the same when defined as a number of grams of protein [50]. Consequently, lower-protein weight-loss diets could preferentially induce negative nitrogen balance due to an absolute decrease in participants’ protein intake.

There is, however, some evidence that protein and amino intake affect health and metabolism differently in males and females [51,52,53,54]. Investigators used data from 27,799 men and 36,875 women aged 45 to 75 from the Japan Public Health Center-Based Prospective Study to investigate the association between a low-carbohydrate diet and the risk of T2D among Japanese men and women, taking into consideration plant and animal sources of protein [55]. Over a 5-year period, 1191 new cases of T2D were reported. Findings indicated that a low-carbohydrate diet, particularly one high in animal protein and fat, was associated with a decreased risk of T2D in women, while men with greater plant fat intake showed lower T2D risk. Here, women showed a significantly increased risk of T2D with higher carbohydrate consumption. Similarly, another prospective cohort study of older Australian men (*n* = 794) showed that total protein intake was associated with increased all-cause mortality, whereas plant protein showed an inverse relationship with all-cause mortality in these men [56], warranting further investigation into the differing impacts of animal-based and plant-based proteins and diets in men and women. It is also unclear whether the amino acid composition of diets high in animal protein affects long-term cardiometabolic outcomes beyond what is induced by atherogenic lipids found in animal foods. 

Some evidence indicates that protein intake affects energy metabolism differently in males and females [51,52,53,54]. Using a randomized cross-over design, investigators used a whole-room calorimeter to determine the effects of dietary macronutrient distribution differences on energy metabolism, satiety, and associated hormones [54]. Ten metabolically healthy, young men were recruited to compare findings with those of a previous study that included only similarly healthy women [57]. Thirty-six-hour, eucaloric diet interventions contained 10% or 30% protein, 30% fat, and the remainder as carbohydrates in the low- and high-protein diets, respectively. Investigators reported that men showed a greater increase in energy expenditure in response to the higher-protein diet, while women showed a greater satiety response to the higher-protein diet.

Particularly in the context of energy-restricted diets, dietary interventions testing the efficacy of higher-protein diets during weight loss show generally better body composition outcomes when women follow a hypocaloric diet higher in protein, rather than carbohydrates, when fat remains constant [45,46,58]. One group tested the effect of a higher-protein diet including 30% of energy from protein at 1.5 g/kg/day compared with a higher-carbohydrate diet restricting protein to 0.8 g/kg/day) in overweight and obese middle-aged women (*n* = 24). Both diets provided consistent fat intake at 30% of energy consumption. During the 10-week dietary intervention period, women consumed food in a laboratory setting for the first 4 weeks, combined with intensive dietary instruction. Women prepared food themselves during the final 6 weeks. After 10 weeks, the women assigned to the carbohydrate intervention had significantly lower fasting blood glucose, which was not observed in the high-protein group [59]. Nonetheless, by showing a greater fat–lean loss ratio, the women in the higher-protein intervention spared fat-free mass compared with women in the carbohydrate group [45]. In lieu of a standard oral glucose tolerance test, the investigators chose to use isocaloric test meals mirroring the assigned intervention diet; hence, carbohydrate load was different during the meal challenge, making meaningful carbohydrate metabolism group comparisons difficult [60]. The same research group later conducted a 12-month study where male and female participants with obesity (*n* = 130) were randomized to similar diet groups [46]. While the protein diet group showed greater fat loss compared with the carbohydrate group, there was no tendency for the protein group to retain more fat-free mass in the mixed-sex trial [46], contrasting with the studies that included only females. Similarly, a 16-week trial comparing the effects of a high-protein vs. lower-protein diet in men and women showed that men lost 2.5 kg fat-free body mass on the high-protein diet, while women retained fat-free mass [42]. On the other hand, a study of leucine supplementation during a controlled 8-week diet showed that leucine better supported fat-free mass retention in male participants [44]. 

Green et al. examined the impact of low-protein diets on metabolic health in different strains and sexes of mice [52]. The findings showed that the benefits of a low-protein diet, such as improved glucose tolerance and increased energy expenditure, were influenced by both sex and genetic background. For instance, mirroring results of the human calorimeter study above, male mice on a low-protein diet showed a significant increase in energy expenditure, whereas female mice did not. Additionally, changes in adiposity and insulin sensitivity varied significantly between male and female mice, with males showing a greater reduction in adiposity and females displaying improved insulin sensitivity. These findings highlight the importance of considering sex and genetic background in dietary interventions for metabolic health. Several compelling studies related to protein and sex using pre-clinical models have been published in recent years [41,47,48,53,61,62].

**Table 1:** Macronutrient Ratios and Quality.

Author	Study Details	Participants	Findings	Limitations
Schulze [26]	Prospective cohort study, 8 to 10-year follow-up. Evaluated whether dietary carbohydrate content or substitution for other macronutrients predicted T2D.	*n* = 25,067 Primarily middle-aged adults without T2D at baseline.	Replacing 5% protein energy with carbohydrate reduced T2D risk in men only.	Carbohydrate quality not considered in substitution modeling.
Vitale et al. [29]	12-week clinical trial (parallel, randomized). Intervention: low- or high-glycemic index Mediterranean diet; both diets included carbohydrates (270 g/day) and fiber (35 g/day). Participants received dietary education and diet-specific carbohydrate foods.	*n* = 156 Middle-aged and older adults. Overweight and obese—WC > 102 cm for men and >88 cm for women. At least one additional feature of the metabolic syndrome.	↑ 8 h baseline-adjusted mean plasma glucose concentrations following meal tolerance tests in women assigned high-GI vs. low-GI diet and within high-GI group before and after intervention. Men showed no differences.	Higher baseline WC, fasting BG, HOMA-IR, SBP, and triglycerides and lower HDL-C in men compared with women.
Nanri et al. [31]	Prospective cohort study, 5-year follow-up. Evaluated whether intake of starch foods predicted T2D incidence.	*n* = 59,288 Middle-aged and older adults without T2D at baseline.	↑ incidence of T2D in women consuming ≥ 3 bowls of white rice each day, not observed in men.	Diet measured once at baseline; T2D diagnosis based on self-report.
Kanehara et al. [32]	Prospective cohort study, 5-year follow-up. Evaluated whether intake of refined carbohydrate foods predicted T2D incidence.	*n* = 64,677 Mostly healthy middle-aged and older adults.	↑ incidence of type 2 in women with high intake of total sugar, fructose, and starch, men showed no association.	T2D diagnosis based on self-report.
Nanri et al. [55]	Prospective cohort study, 5-year follow-up. Evaluated effect of plant and animal protein intake on T2D incidence in men and women.	*n* = 64,674 Middle aged to older adults without history of T2D or other major chronic diseases.	↓ risk of T2D in women consuming low-carbohydrate, high-animal protein diet, but not in men.	T2D diagnosis based on self-report. Dietary intake was only measured once; did not include data on covariates such as physical activity and SES.
Kovar et al. [35]	Clinical trial (crossover, non-randomized). Intervention: participants consumed low-fat diet followed by high-fat, high-cholesterol diet for 2 weeks each.	*n* = 10 Healthy young adults, normal BMI.	↑ in LDL-C and HDL-C cholesterol more pronounced in women vs. Men. ↑ postprandial triglyceridemia in men on high-fat diet.	Diet order not randomized and no washout period; diets were self-selected and compliance was based on self-report.
Niinikoski et al. [37]	Clinical trial (randomized, parallel). Intervention: infants aged 7 to 36 months assigned to typical diet or diet low in cholesterol and saturated fat with energy contributions of 10–15% protein, 50–60% carbohydrates, and 30–35% fat. Both groups were visited 10 times by the study’s counseling team.	*n* = 1062 healthy infants enrolling at 7 months of age, continuing until 36 months.	Significant sex interaction for baseline-adjusted mean cholesterol; percent difference between control and intervention was +6% in boys, +3% in girls.	Food intake and serving size parent-reported; parents and daycare center providers recorded food differently; unable to accurately obtain nutrient intake information for children that were breastfed < 13 months.
Westerterp-Plantenga et al. [54]	Clinical trial (crossover, randomized). Intervention: during each experimental period, participants spent 3 days in a whole-room calorimeter. Adequate-protein diet: energy provided was 10% protein, 60% carbohydrates, and 30% fat High-protein diet: energy provided was 30% protein, 40% carbohydrates, 30% fat.	*n* = 10 Healthy young adults.	High-protein diet promoted ↑ satiety in women only. High-protein diet promoted greater ↑ energy expenditure and substrate oxidation in men compared with women.	Analysis of two separate trials rather than designed as one single trial.
Farnsworth et al. [42]	Clinical trial (parallel, randomized). Intervention: diets were energy-restricted for first 12 weeks followed by 4 weeks of energy balance. High-protein: energy provided was 27% protein, 44% carbohydrate, and 29% fat. Standard-protein: energy provided was 16% protein, 57% carbohydrate, and 27% fat. 60% of energy intake was met by supplied foods.	*n* = 57 Young and middle-aged adults with BMI ≥ 27. High fasting insulin concentration but otherwise generally healthy.	Men lost 2.5 kg fat-free mass on the high-protein diet, while women remained the same.	In total, 75% of participants were women; bodyweight and fasting plasma glucose concentrations were significantly greater in the men than in the women at baseline.
Pathak et al. [44]	Clinical trial (randomized, parallel). Intervention: prescribed 8-week energy restricted to estimated 75% of energy needs with limited animal protein intake, leucine (3 g/day), or placebo.	*n* = 30 Young and middle-aged adults with elevated WC and meeting one additional metabolic syndrome criterion.	Males in the leucine group had a higher fat-free mass compared with placebo after 8 weeks, while females had no increase.	Leucine dose not adjusted for bodyweight; many fewer men than women.

T2D: type 2 diabetes; ↑: higher; ↓: lower; WC: waist circumference; GI: glycemic index; BG: blood glucose; HOMA-IR: homeostatic model assessment of insulin resistance; SBP: systolic blood pressure; LDL-C: low-density lipoprotein cholesterol; HDL-C: high-density lipoprotein cholesterol.

## 4. Mediterranean Diet

While observational studies and clinical trials have demonstrated the potential of a Mediterranean diet to improve biomarkers of metabolic health and reduce risk of developing T2D [63], some studies have also investigated whether men and women respond differently to this diet (Table 2). Bedard et al. conducted a 4-week Mediterranean diet study where all meals were provided to 70 overweight and obese participants, including 38 men and 32 premenopausal women aged 24 to 53 years. The primary aim of the study was to document sex differences in the impact of the Mediterranean diet on cardiometabolic outcomes. After following the 4-week diet, men and women showed similar reductions in fasting blood glucose, homeostasis model assessment of insulin resistance (HOMA-IR), and circulating lipids [59]. The results of a 180 min oral glucose tolerance test (OGTT), however, showed that only men had a significant reduction in plasma insulin incremental area under the curve (iAUC), which included a significant sex-by-time interaction effect. These findings suggest that men have increased peripheral insulin sensitivity in response to a Mediterranean diet intervention. Differences in adipose tissue profiles between men and women may explain the increased insulin sensitivity found in men. Women typically have more subcutaneous fat than men, while men have more visceral fat compared with women [64]. Decreased visceral adipose tissue is associated with improvements in insulin sensitivity and it is possible that men lose more visceral fat compared with women. Additionally, men are known to have higher levels of intramyocellular lipids (IMCL), lipids within skeletal myocytes, compared with women of similar BMI [65]. Increased IMCL is linked with insulin resistance pathology [66,67] and therefore, it is possible that oxidation of these lipids may explain the greater insulin sensitivity improvements experienced by men at the same weight-loss point. This possibility is supported by a significant increase in the Cederholm index, a measure of peripheral insulin sensitivity, in men only [63]. 

In another investigation of the Mediterranean diet, Leblanc et al. studied sex-specific differences in response to a 12-week intervention in middle-aged, overweight, and obese participants meeting at least one metabolic syndrome criterion and having elevated circulating atherogenic lipoproteins (*n* = 64 men and 59 premenopausal women) [68]. In contrast to the controlled feeding study above, these participants self-selected the foods in their diets with support from group, one-on-one, and phone counseling sessions based on motivational interviewing. There were no differences in Mediterranean diet score at baseline, after the 12-week diet, or at 6-month follow-up between men and women, and both sexes reverted to baseline Mediterranean diet scores at the 6-month follow-up. Despite similar adherence to the diet, only men showed reductions in bodyweight, body fat %, low-density lipoprotein cholesterol (LDL-C), and diastolic blood pressure at the conclusion of the study or 6 months post-intervention. While both sexes showed a significant reduction in waist circumference immediately after the dietary intervention, only men maintained a smaller waist circumference through the 6-month follow-up. Though the Mediterranean diet score did not significantly vary between the sexes at any point, and greater weight loss in males may have been explained in part by sex-based differences in metabolic rate, as men showed greater long-term reductions in energy intake and increases in fiber intake. It is unclear whether the study’s outcomes resulted from these sustained dietary changes or intrinsic metabolic differences between the sexes.

Jennings et al. conducted a 12-month randomized controlled trial (RCT) of a Mediterranean-style diet in 1128 healthy participants aged 65 to 79 years from locations across Europe to determine how this diet affected vascular health [69]. The Mediterranean diet group received diet counseling and were provided with wholegrain pasta, extra virgin olive oil, low-fat low-salt cheese, high-polyunsaturated fat margarine, and vitamin D supplements (10 µg/day) to support compliance with the intervention, while the control group followed their habitual diet with general dietary guidance. After a year on the Mediterranean diet, only males showed a significant reduction in systolic blood pressure (SBP; about 9 mm Hg less than controls) and pulse pressure (about 6 mm Hg less than controls). In contrast, in a subset of participants (*n* = 225), only women showed a significant decrease in augmentation index, a measure of arterial stiffness, following the intervention. Moreover, a report from the 4-week controlled feeding study detailed above also described reduced systolic blood pressure in men only [31]. When these results are taken together, men tend to show greater improvements in cardiometabolic outcomes after following a Mediterranean-style diet for up to 12 months. 

**Table 2:** Mediterranean Diet.

Author	Study Details	Participants	Findings	Limitations
Bedard et al. [59]	Clinical trial (single arm). Intervention: participants provided 4-week Mediterranean diet after 4-week controlled diet run-in.	*n* = 70 Young and middle-aged adults; premenopausal women; elevated LDL-C or total cholesterol–HDL-C ratio; at least one factor for metabolic disease	2 h post OGTT insulin concentrations decreased more in men; fasting total cholesterol–HDL-C ratio, LDL-C–HDL-C ratio, apoA2 concentrations, and systolic blood pressure decreased in men only.	No control diet employed.
Leblanc et al. [68]	Clinical trial (single arm). Intervention: self-selected 12-week Mediterranean diet with MI counseling; 35% fat, 45% carbohydrate, 18% protein, 2% alcohol (approximate).	*n* = 103 Young and middle-aged adults; pre-menopausal women; met one criterion for metabolic syndrome; slightly elevated LDL-C and mostly healthy otherwise.	↓ lipids, % body fat, weight, and diastolic BP in men only.	Decreases in energy intake and saturated fat, and increase in fiber, higher in men over intervention and at 6-month follow-up compared with women.
Jennings et al. [69]	Clinical trial (parallel, randomized). Intervention: assignment to usual diet or Mediterranean diet for 12 months. Wholegrain pasta, extra virgin olive oil, low-fat low-salt cheese, high-polyunsaturated fat margarine, and vitamin D supplements provided to Mediterranean diet arm.	*n* = 1128 Older adults.	↓ systolic blood pressure and pulse pressure in men only. ↓ urinary 24 h sodium in men only. ↓ arterial stiffness (augmentation index) in women only.	While appropriate covariates were applied in modeling, it is not clear whether anti-hypertensive medication use or baseline diet differed between men and women.

LDL-C: low-density lipoprotein cholesterol; HDL-C: high-density lipoprotein cholesterol; OGTT: oral glucose tolerance test; MI: motivational interviewing; ↓: lower; BP: blood pressure.

## 5. Western Diets

The Western diet pattern is characterized by chronic consumption of meats, high-fat dairy, processed foods, fried foods, and sweets. The Western diet is associated with chronic inflammation [70] and a sequela of metabolic disorders including obesity [71], T2D [72], and CVD [73]. Evidence from human and animal studies indicates that the effects of a Western-style diet on the inflammatory response, postprandial lipid profile [35], glucose tolerance [74], weight gain [74], and metabolic enzyme mRNA expression are sex-specific [75,76].

Some studies have investigated whether responses to dietary changes shifting away from a Western or more processed diet affect men and women differently (Table 3). A prospective study examined sex differences in T2D risk factors among participants in a diabetes prevention program [77]. While intensive lifestyle modification significantly prevented conversion from prediabetes to T2D overall, men achieved more intensive lifestyle intervention goals than women throughout the intervention but had a similar T2D incidence. Over the first year of the diabetes prevention program, weight loss of 3–7% body weight led to greater improvements in certain risk factors for T2D in men compared with women, and weight loss exceeding 7% showed similar trends. Despite these differences, baseline risk factors in men may have masked a potential sex disparity in incident T2D.

Similarly, men may respond more favorably to a diet that is reduced in atherogenic dietary lipids compared with a Western diet. In one cross-over study, middle-aged to older men (*n* = 19) and postmenopausal women (*n* = 14) with LDL-C concentrations of ≥4.14 mmol/L were provided a diet consistent with therapeutic lifestyle changes (TLC, diet: 26% of energy as fat, 4% as saturated fat, and 45 mg cholesterol/4.2 MJ), which was compared with a typical Western diet (35% of energy as fat, 14% as saturated fat, and 147 mg cholesterol/4.2 MJ) [78]. Following 6 weeks of TLC, men generally showed significantly more pronounced changes in both fasting and post-prandial lipids. The total cholesterol to high-density lipoprotein cholesterol ratio was reduced by 5% in men yet increased by 5% in women. Additional research has recapitulated findings of a low-fat/high-carbohydrate diet inducing more improved lipid profiles in men [79,80]; however, other studies have shown no differences between the sexes [81,82]. 

Additional evidence indicates that women may be especially susceptible to cardiovascular disease when consuming diets high in refined plant foods, i.e., white grains, sugar-sweetened beverages, and other processed foods. Use of diet indices is a common method for measuring diet quality and adherence to healthy dietary patterns [83]. These tools allow comparative assessment of diet quality, which is generally applicable across most population categories. Using food-frequency questionnaires from the Nurses’ Health Study 1 and 2 (NHS 1 and NHS2; female participants) and the Health Professionals Follow-Up Study (male participants), study authors created three plant-food index scores to define plant-based diets (plant-based diet index; PDI), healthy plant-based diets (hPDI), and unhealthy plant-based diets (uPDI) [84,85]. All plant-based foods contribute positively to the PDI, only healthful/whole plant foods contribute positively to the hPDI, and unhealthful plant foods contribute positively to the uPDI. Any dietary component that does not “fit” the defined diet contributes negative scores, i.e., animal foods for all versions of the PDI, processed plant foods for the hPDI, and whole plant foods for the uPDI. The authors then modeled how compliance with each of the dietary patterns is associated with risk of developing coronary heart disease (CHD) later in life [84]. Based on fully adjusted multivariable models, women in the highest versus lowest deciles of the uPDI were at a 49% (NHS1) and 77% (NHS2) greater risk of CHD, while male counterparts showed a 21% increased risk for CHD. Though the aggregated data analysis showed an inverse association between hPDI and CHD and a positive association with CHD for uPDI, the risk of developing CHD while following a dietary pattern high in refined carbohydrates and fats appeared greater in women. 

DeGroef et al. investigated sexual dimorphism in metabolic responses to a Western diet in fruit flies, utilizing standard diets supplemented with up to 30% sugar and 30% coconut oil [76]. While female flies on all diets containing added sugar gained more weight compared with females consuming a standard diet, no such weight differences were observed in the male flies with added sugar or fat. That said, male flies consuming any diet with added fat showed greater body triacylglycerol (TAG) storage compared with male flies on the standard diet, but dietary fat-induced TAG differences were attenuated in female flies. Female flies displayed a twofold higher glycogen concentration compared with males under normal conditions and much more diet-associated variability in glycogen storage compared with males. In females, most groups receiving fat-supplemented diets showed a significant decrease in glycogen content. On the other hand, sugar supplementation alone induced substantially higher glycogen storage in female flies, a response not observed in males. Perhaps the female propensity to store, rather than metabolize, glucose could reduce glycogen disposal capacity at later eating occasions and contribute to glucose intolerance with chronically high consumption of refined carbohydrates. Moreover, mRNA abundance of several genes encoding proteins critical for lipid and carbohydrate metabolism showed sexual dimorphism in their expression patterns. That study demonstrated that a Western-style diet induced metabolic changes and metabolism-related gene expression in a sex-specific manner in fruit flies, shedding light on potential metabolic mechanisms underlying sex–diet interactions.

**Table 3:** Western Diet.

Author	Study Details	Participants	Findings	Limitations
Perreault et al. [77]	Clinical trial (secondary analysis of DPP intervention and placebo arms [86]). Intervention: Reduced-calorie, low-fat diet; 150 min/week moderate physical activity; 1-year follow-up	*n* = 3000 Age ≥ 25 years, BMI ≥ 24, met diagnostic criteria for prediabetes.	3–7% weight loss showed greater reduction in 2 h OGTT glucose/insulin and insulin resistance in men than in women; >7% weight loss showed greater reduction in 2 h OGTT and hemoglobin A1C in men compared with women.	Men had a greater load of baseline risk factors compared with women; no supervised intervention.
Li et al. [78]	Clinical trial (crossover, non-randomized). Intervention: 6-week American-style diet was followed by 2–7-week washout and the TLC diet for 6 weeks. TLC diet: energy distribution of 16% protein, 58% carbohydrate, 25% fat, 4% saturated fat, and 45 mg cholesterol/4.2 MJ. American diet: energy distribution of 15% protein, 49% carbohydrate, 35% fat, 14% saturated fat, and 147 mg cholesterol/4.2 MJ.	*n* = 33 Middle-aged and older adults with moderate hypercholesterolemia but otherwise healthy; postmenopausal women.	↑ fasting triglycerides following TLC in women but not in men; ↓ in postprandial triglyceride concentration in men but not women; total cholesterol–HDL-C ratio in men decreased by 5%, while women showed a 5% increase.	Men and women not evaluated in same statistical model; only within-sex group *t*-tests were used.
Satija et al. [84]	Prospective cohort study, 22- and 26-year follow up. Compared outcomes between lowest and highest deciles of three plant-based diet indices: PDI, hPDI, and uPDI.	*n* = 209,928 Mostly healthy middle-aged and older adult health professionals.	↑ CHD with high uPDI score appeared greater in women compared with men (hazard ratio 1.6 vs. 1.18 in top decile, for women and men, respectively).	No direct statistical comparison between men and women; self-reported data.

DPP: diabetes prevention program; BMI: body mass index; OGTT: oral glucose tolerance test; TLC diet: therapeutic lifestyle changes diet; HDL-C: high-density lipoprotein cholesterol; ↑: higher; ↓: lower.

## 6. Very Low-Calorie Diets

Recent research has increasingly considered the influence of sex as a biological variable in response to medical VLCDs (Table 4). A large multinational study including 2020 overweight individuals with prediabetes (PREVIEW lifestyle intervention study) examined sex-specific responses to a VLCD (810 calories/day) [87]. While men and women experienced similar improvements in insulin resistance, men lost 16% more weight after 8 weeks on an energy-restricted diet, which could be explained by a difference in absolute energy deficit between the sexes, given the same energy prescription for all participants. That said, women experienced significantly greater reductions in fat-free mass, hip circumference, and high-density lipoprotein cholesterol (HDL-C) concentration. Women were also significantly more likely to experience adverse reactions during and immediately following the low-energy diet, including constipation, diarrhea GI symptoms (nausea, pain, vomiting), sore throat, headaches and migraines, muscular weakness and pain, hair loss, and infections.

Investigations into the effects of low-energy diets have revealed sexual dimorphism in weight loss and cardiometabolic risk factors. Trouwborst and colleagues conducted a randomized controlled trial where 782 participants with BMI ≥ 25 (65% women) consumed 800 kcal/day for 8 weeks and were then randomized to either a control or one of four different *ad libitum* diets that varied in protein content and glycemic index [88]. Men experienced greater weight loss during the low-calorie diet period compared with the women but regained more weight during the follow-up period. Men also showed more pronounced improvements in various cardiometabolic risk factors, such as insulin sensitivity, cholesterol levels, and blood pressure, even after adjusting for weight change. Conversely, women demonstrated smaller rebounds in HDL-C, triacylglycerol, and diacylglycerol concentrations during the weight maintenance phase, independent of weight changes. These differences suggest that metabolic responses to weight loss and maintenance are influenced by sex-specific factors, which may include body fat distribution, baseline metabolic status, and absolute energy deficit induced by the intervention.

Tremblay et al. examined the profiles of individuals who had been successful or unsuccessful in losing weight on a calorie-restricted diet [89]. The study found that approximately 10% of women and 8% of males were unsuccessful at losing weight on a VLCD and that males experienced greater reductions in fat mass, body fat percentage, and waist circumference compared with women. Additionally, the differences between unsuccessful and successful responders were more pronounced in men. However, men weighed more at baseline, and men who were successful or unsuccessful had greater average energy deficits (1659 and 815 kcal) compared with their female counterparts (1299 and 656 kcal), which may explain these differences. Researchers point to less favorable changes in appetite and hunger sensations among unsuccessful responders and suggest that these individuals display a “behavioral vulnerability” which may reduce their ability to lose weight during a weight-loss program. 

In recent years, forms of intermittent fasting (IF), for example, time-restricted feeding and alternate-day fasting, have been studied more extensively. Potential for sexual dimorphism in IF was recently reviewed in detail by Rius-Bonet et al. [90]. The authors posited that due to women’s higher lipolytic rates during fasting, women may achieve similar health benefits from shorter fasting windows compared with men. Readers are directed to this article for an extended review and discussion on the topic of sexual dimorphism in IF and the role of menopause in these interactions. 

**Table 4:** Very Low-Calorie Diet.

Author	Diet Type and Composition	Participants	Findings	Limitations
Christensen et al. [87]	Clinical trial (single arm). Intervention: 810 kcal, 85 g protein, 5 g essential fatty acids, and 13 g fiber each day	*n* = 2020 Adults with prediabetes, BMI ≥ 25.	Men showed more reduced metabolic syndrome Z-scores and fat mass compared with women even after adjusting for weight loss; women lost twice as much fat-free mass as men.	Dropout rates varied between sites and were lower among men; significantly greater relative energy deficit for men.
Trouwborst et al. [88]	Clinical trial (secondary analysis). Intervention: 8-week 800 kcal/day, followed by 6-month phase with *ad libitum* diet.	*n* = 555 Overweight and obese adults under 65 years of age with fasting blood glucose concentrations < 6.1 mmol/L.	Men showed greater reductions in weight, insulin sensitivity, TAG, and LDL-C after adjusting for weight loss, but weight and cardiometabolic indicators rebounded more in the follow-up period compared with women.	High dropout rate; did not report body composition measures; 5 experimental arms of *ad libitum* phase after VLCD may have affected follow-up outcomes; significantly greater relative energy deficit for men.
Tremblay et al. [89]	Clinical trial (secondary analysis of VLCD diet phase). Intervention: 800 kcal/day, 15–20% fat, 35–40% protein, 45–50% carbohydrate for 8 weeks.	Overweight and obese adults with impaired glucose tolerance.	Males showed greater reductions in fat mass, body fat percentage, and waist circumference compared with women in the 8-week weight-loss phase.	Significantly greater relative energy deficit for men.

BMI: body mass index; TAG: triacylglycerol; LDL-C: low-density lipoprotein cholesterol; HDL-C: high-density lipoprotein cholesterol; VLCD: very low-calorie diet.

## 7. Limitations

Many studies examining sex-specific response to diet do not control for or consider the role of sex hormones and menopausal status in relation to cardiometabolic outcomes. Sex hormones, such as estrogen and testosterone, are known to influence metabolism and nutrient utilization. Exogenous sources of sex hormones or highly variable endogenous levels are likely to affect metabolic outcomes. Moreover, hormonal fluctuations throughout the menstrual cycle can affect appetite [91,92,93], food intake [94], nutrient metabolism [95], and circulating concentrations of many metabolites and micronutrients [96]. The process of menopause induces changes in gut and sex hormones [97] and circadian rhythms [98], in turn affecting nutrient metabolism and disease risk. Parity as well as age at menarche and menopause also impact metabolic traits in women [99]. 

Additionally, many studies include primarily middle-aged adults, so less is known about how sex-specific dietary responses may vary across different life stages. Physical activity significantly alters nutrient metabolism, yet few studies integrate this variable into their analyses to capture its interaction with diet and sex. Addressing these gaps in future research will advance our understanding of how to tailor dietary recommendations to optimize health outcomes for both men and women across the lifespan.

The studies included here had great variability in design and heterogeneity in dietary interventions or definitions. They were also mostly secondary analyses of previously completed studies. The findings presented here should be interpreted with caution, as they may have been influenced by the limitations of the original study designs and potential biases inherent in secondary data analysis and narrative reviews.

## 8. Conclusions

Sexual dimorphism significantly influences nutrient requirements, metabolic processes, and disease susceptibility and is driven by factors such as sex hormones, body fat distribution, and many other factors [12]. These biological differences contribute to divergent metabolic responses to dietary components and patterns, highlighting the need for sex-specific considerations in nutrition recommendations; however, many studies do not include both sexes [100]. Existing research indicates that women are potentially more susceptible to T2D with chronic refined carbohydrate intake, whereas men may be more susceptible to chronic disease when chronically consuming high-fat diets, particularly those high in animal products. These findings demonstrate the need to develop sex-specific dietary strategies for the prevention of metabolic diseases like T2D. The field of nutrition needs powered clinical trials to test sex-specific responses to various dietary patterns and macronutrients, while also considering the role of physical activity and additional biological, psychosocial, and socioecological factors to facilitate development of precision dietary guidelines.

## References

[1] Micha R., Peñalvo J.L., Cudhea F., Imamura F., Rehm C.D., Mozaffarian D. (2017). Association Between Dietary Factors and Mortality From Heart Disease, Stroke, and Type 2 Diabetes in the United States. JAMA.

[2] Beigrezaei S., Ghiasvand R., Feizi A., Iraj B. (2019). Relationship between Dietary Patterns and Incidence of Type 2 Diabetes. Int. J. Prev. Med..

[3] Fung T.T., Schulze M., Manson J.E., Willett W.C., Hu F.B. (2004). Dietary patterns, meat intake, and the risk of type 2 diabetes in women. Arch. Intern. Med..

[4] McEvoy C.T., Cardwell C.R., Woodside J.V., Young I.S., Hunter S.J., McKinley M.C. (2014). A posteriori dietary patterns are related to risk of type 2 diabetes: Findings from a systematic review and meta-analysis. J. Acad. Nutr. Diet..

[5] Mihaylova M.M., Chaix A., Delibegovic M., Ramsey J.J., Bass J., Melkani G., Singh R., Chen Z., Ja W.W., Shirasu-Hiza M. (2023). When a calorie is not just a calorie: Diet quality and timing as mediators of metabolism and healthy aging. Cell Metab..

[6] van Dam R.M., Rimm E.B., Willett W.C., Stampfer M.J., Hu F.B. (2002). Dietary patterns and risk for type 2 diabetes mellitus in U.S. men. Ann. Intern. Med..

[7] Luo Y., Wang J., Sun L., Gu W., Zong G., Song B., Shen C., Zhou P., Chen Y., Wu Y. (2022). Isocaloric-restricted Mediterranean Diet and Chinese Diets High or Low in Plants in Adults With Prediabetes. J. Clin. Endocrinol. Metab..

[8] Guasch-Ferré M., Santos J.L., Martínez-González M.A., Clish C.B., Razquin C., Wang D., Liang L., Li J., Dennis C., Corella D. (2020). Glycolysis/gluconeogenesis- and tricarboxylic acid cycle-related metabolites, Mediterranean diet, and type 2 diabetes. Am. J. Clin. Nutr..

[9] Sellahewa L., Khan C., Lakkunarajah S., Idris I. (2017). A Systematic Review of Evidence on the Use of Very Low Calorie Diets in People with Diabetes. Curr. Diabetes Rev..

[10] Umphonsathien M., Rattanasian P., Lokattachariya S., Suansawang W., Boonyasuppayakorn K., Khovidhunkit W. (2022). Effects of intermittent very-low calorie diet on glycemic control and cardiovascular risk factors in obese patients with type 2 diabetes mellitus: A randomized controlled trial. J. Diabetes Investig..

[11] Yuan X., Wang J., Yang S., Gao M., Cao L., Li X., Hong D., Tian S., Sun C. (2022). Effect of Intermittent Fasting Diet on Glucose and Lipid Metabolism and Insulin Resistance in Patients with Impaired Glucose and Lipid Metabolism: A Systematic Review and Meta-Analysis. Int. J. Endocrinol..

[12] Pontifex M.G., Vauzour D., Muller M. (2024). Sexual dimorphism in the context of nutrition and health. Proc. Nutr. Soc..

[13] Moschonis G., Papandreou D., Mavrogianni C., Giannopoulou A., Damianidi L., Malindretos P., Lionis C., Chrousos G.P., Manios Y. (2013). Association of iron depletion with menstruation and dietary intake indices in pubertal girls: The healthy growth study. BioMed Res. Int..

[14] Zore T., Palafox M., Reue K. (2018). Sex differences in obesity, lipid metabolism, and inflammation-A role for the sex chromosomes?. Mol. Metab..

[15] Giagulli V.A., Castellana M., Pelusi C., Triggiani V. (2019). Androgens, Body Composition, and Their Metabolism Based on Sex. Front. Horm. Res..

[16] Krishnan S., Tryon R.R., Horn W.F., Welch L., Keim N.L. (2016). Estradiol, SHBG and leptin interplay with food craving and intake across the menstrual cycle. Physiol. Behav..

[17] Morford J., Mauvais-Jarvis F. (2016). Sex differences in the effects of androgens acting in the central nervous system on metabolism. Dialogues Clin. Neurosci..

[18] D’Abbondanza M., Ministrini S., Pucci G., Nulli Migliola E., Martorelli E.E., Gandolfo V., Siepi D., Lupattelli G., Vaudo G. (2020). Very Low-Carbohydrate Ketogenic Diet for the Treatment of Severe Obesity and Associated Non-Alcoholic Fatty Liver Disease: The Role of Sex Differences. Nutrients.

[19] Prentice A. (2021). Sex differences in requirements for micronutrients across the lifecourse. Proc. Nutr. Soc..

[20] Tottman A.C., Oliver C.J., Alsweiler J.M., Cormack B.E. (2021). Do preterm girls need different nutrition to preterm boys? Sex-specific nutrition for the preterm infant. Pediatr. Res..

[21] O’Neil A., Scovelle A.J., Milner A.J., Kavanagh A. (2018). Gender/Sex as a Social Determinant of Cardiovascular Risk. Circulation.

[22] Clegg D.J., Mauvais-Jarvis F. (2018). An integrated view of sex differences in metabolic physiology and disease. Mol. Metab..

[23] Bailey R.L., Dog T.L., Smith-Ryan A.E., Das S.K., Baker F.C., Madak-Erdogan Z., Hammond B.R., Sesso H.D., Eapen A., Mitmesser S.H. (2022). Sex Differences Across the Life Course: A Focus On Unique Nutritional and Health Considerations among Women. J. Nutr..

[24] Farag M.A., Hamouda S., Gomaa S., Agboluaje A.A., Hariri M.L.M., Yousof S.M. (2021). Dietary Micronutrients from Zygote to Senility: Updated Review of Minerals’ Role and Orchestration in Human Nutrition throughout Life Cycle with Sex Differences. Nutrients.

[25] Michael R.M.a.S.J.M. (2023). Sex differences in healthspan and lifespan responses to geroprotective dietary interventions in preclinical models. Curr. Opin. Physiol..

[26] Schulze M.B., Schulz M., Heidemann C., Schienkiewitz A., Hoffmann K., Boeing H. (2008). Carbohydrate intake and incidence of type 2 diabetes in the European Prospective Investigation into Cancer and Nutrition (EPIC)-Potsdam Study. Br. J. Nutr..

[27] Clapp J.F., Lopez B. (2007). Low-versus high-glycemic index diets in women: Effects on caloric requirement, substrate utilization and insulin sensitivity. Metab. Syndr. Relat. Disord..

[28] Beulens J.W., de Bruijne L.M., Stolk R.P., Peeters P.H., Bots M.L., Grobbee D.E., van der Schouw Y.T. (2007). High dietary glycemic load and glycemic index increase risk of cardiovascular disease among middle-aged women: A population-based follow-up study. J. Am. Coll. Cardiol..

[29] Vitale M., Costabile G., Bergia R.E., Hjorth T., Campbell W.W., Landberg R., Riccardi G., Giacco R. (2023). The effects of Mediterranean diets with low or high glycemic index on plasma glucose and insulin profiles are different in adult men and women: Data from MEDGI-Carb randomized clinical trial. Clin. Nutr..

[30] Livesey G., Taylor R., Livesey H.F., Buyken A.E., Jenkins D.J.A., Augustin L.S.A., Sievenpiper J.L., Barclay A.W., Liu S., Wolever T.M.S. (2019). Dietary Glycemic Index and Load and the Risk of Type 2 Diabetes: A Systematic Review and Updated Meta-Analyses of Prospective Cohort Studies. Nutrients.

[31] Nanri A., Mizoue T., Noda M., Takahashi Y., Kato M., Inoue M., Tsugane S., Group J.P.H.C.-b.P.S. (2010). Rice intake and type 2 diabetes in Japanese men and women: The Japan Public Health Center-based Prospective Study. Am. J. Clin. Nutr..

[32] Kanehara R., Goto A., Sawada N., Mizoue T., Noda M., Hida A., Iwasaki M., Tsugane S. (2022). Association between sugar and starch intakes and type 2 diabetes risk in middle-aged adults in a prospective cohort study. Eur. J. Clin. Nutr..

[33] Ren G., Qi J., Zou Y. (2021). Association between intake of white rice and incident type 2 diabetes—An updated meta-analysis. Diabetes Res. Clin. Pract..

[34] Hosseini-Esfahani F., Beheshti N., Nematollahi A., Koochakpoor G., Verij-Kazemi S., Mirmiran P., Azizi F. (2023). The association between dietary acrylamide intake and the risk of type 2 diabetes incidence in the Tehran lipid and glucose study. Sci. Rep..

[35] Kovar J., Poledne R. (2000). Sex differences in the response of postprandial lipemia to a change from a low-fat low-cholesterol diet to a high-fat high-cholesterol diet. Physiol. Res..

[36] Palmisano B.T., Zhu L., Stafford J.M. (2017). Role of Estrogens in the Regulation of Liver Lipid Metabolism. Adv. Exp. Med. Biol..

[37] Niinikoski H., Viikari J., Rönnemaa T., Lapinleimu H., Jokinen E., Salo P., Seppänen R., Leino A., Tuominen J., Välimäki I. (1996). Prospective randomized trial of low-saturated-fat, low-cholesterol diet during the first 3 years of life. The STRIP baby project. Circulation.

[38] Estrany M.E., Proenza A.M., Gianotti M., Llado I. (2013). High-fat diet feeding induces sex-dependent changes in inflammatory and insulin sensitivity profiles of rat adipose tissue. Cell Biochem. Funct..

[39] Huang K.P., Ronveaux C.C., Knotts T.A., Rutkowsky J.R., Ramsey J.J., Raybould H.E. (2020). Sex differences in response to short-term high fat diet in mice. Physiol. Behav..

[40] Elzinga S.E., Savelieff M.G., O’Brien P.D., Mendelson F.E., Hayes J.M., Feldman E.L. (2021). Sex differences in insulin resistance, but not peripheral neuropathy, in a diet-induced prediabetes mouse model. Dis. Model. Mech..

[41] Solon-Biet S.M., Cogger V.C., Pulpitel T., Wahl D., Clark X., Bagley E., Gregoriou G.C., Senior A.M., Wang Q.P., Brandon A.E. (2019). Branched chain amino acids impact health and lifespan indirectly via amino acid balance and appetite control. Nat. Metab..

[42] Farnsworth E., Luscombe N.D., Noakes M., Wittert G., Argyiou E., Clifton P.M. (2003). Effect of a high-protein, energy-restricted diet on body composition, glycemic control, and lipid concentrations in overweight and obese hyperinsulinemic men and women. Am. J. Clin. Nutr..

[43] Pasiakos S.M., Mettel J.B., West K., Lofgren I.E., Fernandez M.L., Koo S.I., Rodriguez N.R. (2008). Maintenance of resting energy expenditure after weight loss in premenopausal women: Potential benefits of a high-protein, reduced-calorie diet. Metabolism.

[44] Pathak K., Zhao Y., Calton E.K., James A.P., Newsholme P., Sherriff J., Soares M.J. (2024). The impact of leucine supplementation on body composition and glucose tolerance following energy restriction: An 8-week RCT in adults at risk of the metabolic syndrome. Eur. J. Clin. Nutr..

[45] Layman D.K., Boileau R.A., Erickson D.J., Painter J.E., Shiue H., Sather C., Christou D.D. (2003). A reduced ratio of dietary carbohydrate to protein improves body composition and blood lipid profiles during weight loss in adult women. J. Nutr..

[46] Layman D.K., Evans E.M., Erickson D., Seyler J., Weber J., Bagshaw D., Griel A., Psota T., Kris-Etherton P. (2009). A moderate-protein diet produces sustained weight loss and long-term changes in body composition and blood lipids in obese adults. J. Nutr..

[47] Yu D., Richardson N.E., Green C.L., Spicer A.B., Murphy M.E., Flores V., Jang C., Kasza I., Nikodemova M., Wakai M.H. (2021). The adverse metabolic effects of branched-chain amino acids are mediated by isoleucine and valine. Cell Metab..

[48] Trautman M.E., Richardson N.E., Lamming D.W. (2022). Protein restriction and branched-chain amino acid restriction promote geroprotective shifts in metabolism. Aging Cell.

[49] Hruby A., Jacques P.F. (2021). Protein Intake and Human Health: Implications of Units of Protein Intake. Adv. Nutr..

[50] Westerterp-Plantenga M.S. (2007). How are normal, high- or low-protein diets defined?. Br. J. Nutr..

[51] Richardson N.E., Konon E.N., Schuster H.S., Mitchell A.T., Boyle C., Rodgers A.C., Finke M., Haider L.R., Yu D., Flores V. (2021). Lifelong restriction of dietary branched-chain amino acids has sex-specific benefits for frailty and lifespan in mice. Nat. Aging.

[52] Green C.L., Pak H.H., Richardson N.E., Flores V., Yu D., Tomasiewicz J.L., Dumas S.N., Kredell K., Fan J.W., Kirsh C. (2022). Sex and genetic background define the metabolic, physiologic, and molecular response to protein restriction. Cell Metab..

[53] Larson K.R., Russo K.A., Fang Y., Mohajerani N., Goodson M.L., Ryan K.K. (2017). Sex Differences in the Hormonal and Metabolic Response to Dietary Protein Dilution. Endocrinology.

[54] Westerterp-Plantenga M.S., Lejeune M.P., Smeets A.J., Luscombe-Marsh N.D. (2009). Sex differences in energy homeostatis following a diet relatively high in protein exchanged with carbohydrate, assessed in a respiration chamber in humans. Physiol. Behav..

[55] Nanri A., Mizoue T., Kurotani K., Goto A., Oba S., Noda M., Sawada N., Tsugane S., Group J.P.H.C.-B.P.S. (2015). Low-carbohydrate diet and type 2 diabetes risk in Japanese men and women: The Japan Public Health Center-Based Prospective Study. PLoS ONE.

[56] Das A., Cumming R., Naganathan V., Blyth F., Couteur D.G.L., Handelsman D.J., Waite L.M., Ribeiro R.V.R., Simpson S.J., Hirani V. (2022). Associations between dietary intake of total protein and sources of protein (plant vs. animal) and risk of all-cause and cause-specific mortality in older Australian men: The Concord Health and Ageing in Men Project. J. Hum. Nutr. Diet..

[57] Lejeune M.P., Westerterp K.R., Adam T.C., Luscombe-Marsh N.D., Westerterp-Plantenga M.S. (2006). Ghrelin and glucagon-like peptide 1 concentrations, 24-h satiety, and energy and substrate metabolism during a high-protein diet and measured in a respiration chamber. Am. J. Clin. Nutr..

[58] Te Morenga L.A., Levers M.T., Williams S.M., Brown R.C., Mann J. (2011). Comparison of high protein and high fiber weight-loss diets in women with risk factors for the metabolic syndrome: A randomized trial. Nutr. J..

[59] Bedard A., Riverin M., Dodin S., Corneau L., Lemieux S. (2012). Sex differences in the impact of the Mediterranean diet on cardiovascular risk profile. Br. J. Nutr..

[60] Layman D.K., Shiue H., Sather C., Erickson D.J., Baum J. (2003). Increased dietary protein modifies glucose and insulin homeostasis in adult women during weight loss. J. Nutr..

[61] MacArthur M.R., Mitchell S.J., Chadaideh K.S., Treviño-Villarreal J.H., Jung J., Kalafut K.C., Reynolds J.S., Mann C.G., Trocha K.M., Tao M. (2022). Multiomics assessment of dietary protein titration reveals altered hepatic glucose utilization. Cell Rep..

[62] Yeh C.Y., Chini L.C.S., Davidson J.W., Garcia G.G., Gallagher M.S., Freichels I.T., Calubag M.F., Rodgers A.C., Green C.L., Babygirija R. (2024). Late-life isoleucine restriction promotes physiological and molecular signatures of healthy aging. bioRxiv.

[63] Salas-Salvadó J., Bulló M., Estruch R., Ros E., Covas M.I., Ibarrola-Jurado N., Corella D., Arós F., Gómez-Gracia E., Ruiz-Gutiérrez V. (2014). Prevention of diabetes with Mediterranean diets: A subgroup analysis of a randomized trial. Ann. Intern. Med..

[64] Bredella M.A. (2017). Sex Differences in Body Composition. Adv. Exp. Med. Biol..

[65] Machann J., Thamer C., Schnoedt B., Stefan N., Stumvoll M., Haring H.U., Claussen C.D., Fritsche A., Schick F. (2005). Age and gender related effects on adipose tissue compartments of subjects with increased risk for type 2 diabetes: A whole body MRI/MRS study. Magma.

[66] Shulman G.I. (2000). Cellular mechanisms of insulin resistance. J. Clin. Investig..

[67] Shulman G.I. (2014). Ectopic fat in insulin resistance, dyslipidemia, and cardiometabolic disease. N. Engl. J. Med..

[68] Leblanc V., Begin C., Hudon A.M., Royer M.M., Corneau L., Dodin S., Lemieux S. (2014). Gender differences in the long-term effects of a nutritional intervention program promoting the Mediterranean diet: Changes in dietary intakes, eating behaviors, anthropometric and metabolic variables. Nutr. J..

[69] Jennings A., Berendsen A.M., de Groot L., Feskens E.J.M., Brzozowska A., Sicinska E., Pietruszka B., Meunier N., Caumon E., Malpuech-Brugère C. (2019). Mediterranean-Style Diet Improves Systolic Blood Pressure and Arterial Stiffness in Older Adults. Hypertension.

[70] Christ A., Lauterbach M., Latz E. (2019). Western Diet and the Immune System: An Inflammatory Connection. Immunity.

[71] Lathigara D., Kaushal D., Wilson R.B. (2023). Molecular Mechanisms of Western Diet-Induced Obesity and Obesity-Related Carcinogenesis-A Narrative Review. Metabolites.

[72] Basiak-Rasała A., Różańska D., Zatońska K. (2019). Food groups in dietary prevention of type 2 diabetes. Rocz. Panstw. Zakl. Hig..

[73] Shi W., Huang X., Schooling C.M., Zhao J.V. (2023). Red meat consumption, cardiovascular diseases, and diabetes: A systematic review and meta-analysis. Eur. Heart J..

[74] Salinero A.E., Robison L.S., Gannon O.J., Riccio D., Mansour F., Abi-Ghanem C., Zuloaga K.L. (2020). Sex-specific effects of high-fat diet on cognitive impairment in a mouse model of VCID. FASEB J..

[75] Arner P., Lithell H., Wahrenberg H., Brönnegard M. (1991). Expression of lipoprotein lipase in different human subcutaneous adipose tissue regions. J. Lipid Res..

[76] De Groef S., Wilms T., Balmand S., Calevro F., Callaerts P. (2021). Sexual Dimorphism in Metabolic Responses to Western Diet in Drosophila melanogaster. Biomolecules.

[77] Perreault L., Ma Y., Dagogo-Jack S., Horton E., Marrero D., Crandall J., Barrett-Connor E. (2008). Sex differences in diabetes risk and the effect of intensive lifestyle modification in the Diabetes Prevention Program. Diabetes Care.

[78] Li Z., Otvos J.D., Lamon-Fava S., Carrasco W.V., Lichtenstein A.H., McNamara J.R., Ordovas J.M., Schaefer E.J. (2003). Men and women differ in lipoprotein response to dietary saturated fat and cholesterol restriction. J. Nutr..

[79] Barnard R.J. (1991). Effects of life-style modification on serum lipids. Arch. Intern. Med..

[80] Tentor J., Harada L.M., Nakamura R.T., Gidlund M., Castilho L.N., Cotta de Faria E. (2006). Sex-dependent variables in the modulation of postalimentary lipemia. Nutrition.

[81] Howard B.V., Hannah J.S., Heiser C.C., Jablonski K.A. (1995). Effects of sex and ethnicity on responses to a low-fat diet: A study of African Americans and whites. Am. J. Clin. Nutr..

[82] Geil P.B., Anderson J.W., Gustafson N.J. (1995). Women and men with hypercholesterolemia respond similarly to an American Heart Association step 1 diet. J. Am. Diet. Assoc..

[83] Martinchik A.N. (2019). Indices of diet quality as a tool for integrated assessment of dietary intake. Vopr. Pitan..

[84] Satija A., Bhupathiraju S.N., Spiegelman D., Chiuve S.E., Manson J.E., Willett W., Rexrode K.M., Rimm E.B., Hu F.B. (2017). Healthful and Unhealthful Plant-Based Diets and the Risk of Coronary Heart Disease in U.S. Adults. J. Am. Coll. Cardiol..

[85] Satija A., Bhupathiraju S.N., Rimm E.B., Spiegelman D., Chiuve S.E., Borgi L., Willett W.C., Manson J.E., Sun Q., Hu F.B. (2016). Plant-Based Dietary Patterns and Incidence of Type 2 Diabetes in US Men and Women: Results from Three Prospective Cohort Studies. PLoS Med..

[86] Group T.D.P.P.R. (1999). The Diabetes Prevention Program. Design and methods for a clinical trial in the prevention of type 2 diabetes. Diabetes Care.

[87] Christensen P., Meinert Larsen T., Westerterp-Plantenga M., Macdonald I., Martinez J.A., Handjiev S., Poppitt S., Hansen S., Ritz C., Astrup A. (2018). Men and women respond differently to rapid weight loss: Metabolic outcomes of a multi-centre intervention study after a low-energy diet in 2500 overweight, individuals with pre-diabetes (PREVIEW). Diabetes Obes. Metab..

[88] Trouwborst I., Goossens G.H., Astrup A., Saris W.H.M., Blaak E.E. (2021). Sexual Dimorphism in Body Weight Loss, Improvements in Cardiometabolic Risk Factors and Maintenance of Beneficial Effects 6 Months after a Low-Calorie Diet: Results from the Randomized Controlled DiOGenes Trial. Nutrients.

[89] Tremblay A., Fogelholm M., Jalo E., Westerterp-Plantenga M.S., Adam T.C., Huttunen-Lenz M., Stratton G., Lam T., Handjieva-Darlenska T., Handjiev S. (2021). What Is the Profile of Overweight Individuals Who Are Unsuccessful Responders to a Low-Energy Diet? A PREVIEW Sub-study. Front. Nutr..

[90] Rius-Bonet J., Macip S., Closa D., Massip-Salcedo M. (2024). Intermittent fasting as a dietary intervention with potential sexually dimorphic health benefits. Nutr. Rev..

[91] Buffenstein R., Poppitt S.D., McDevitt R.M., Prentice A.M. (1995). Food intake and the menstrual cycle: A retrospective analysis, with implications for appetite research. Physiol. Behav..

[92] Van Vugt D.A. (2010). Brain imaging studies of appetite in the context of obesity and the menstrual cycle. Hum. Reprod. Update.

[93] Dye L., Blundell J.E. (1997). Menstrual cycle and appetite control: Implications for weight regulation. Hum. Reprod..

[94] Rogan M.M., Black K.E. (2023). Dietary energy intake across the menstrual cycle: A narrative review. Nutr. Rev..

[95] Benton M.J., Hutchins A.M., Dawes J.J. (2020). Effect of menstrual cycle on resting metabolism: A systematic review and meta-analysis. PLoS ONE.

[96] Draper C.F., Duisters K., Weger B., Chakrabarti A., Harms A.C., Brennan L., Hankemeier T., Goulet L., Konz T., Martin F.P. (2018). Menstrual cycle rhythmicity: Metabolic patterns in healthy women. Sci. Rep..

[97] Sowers M.R., Wildman R.P., Mancuso P., Eyvazzadeh A.D., Karvonen-Gutierrez C.A., Rillamas-Sun E., Jannausch M.L. (2008). Change in adipocytokines and ghrelin with menopause. Maturitas.

[98] Furtado A., Costa D., Lemos M.C., Cavaco J.E., Santos C.R.A., Quintela T. (2023). The impact of biological clock and sex hormones on the risk of disease. Adv. Protein Chem. Struct. Biol..

[99] Clayton G.L., Borges M.C., Lawlor D.A. (2024). The impact of reproductive factors on the metabolic profile of females from menarche to menopause. Nat. Commun..

[100] Song S., Kim S., Lee J.E. (2018). Sex consideration in diet-biomarker-related indices: A systematic review. Public Health Nutr..

